# Supplementation with Milk-Derived Extracellular Vesicles Shapes the Gut Microbiota and Regulates the Transcriptomic Landscape in Experimental Colitis

**DOI:** 10.3390/nu14091808

**Published:** 2022-04-26

**Authors:** Chunmei Du, Kun Wang, Yiguang Zhao, Xuemei Nan, Ruipeng Chen, Suyu Quan, Benhai Xiong

**Affiliations:** 1 State Key Laboratory of Animal Nutrition, Institute of Animal Sciences, Chinese Academy of Agricultural Sciences, Beijing 100193, China; duchunmeim@163.com (C.D.); cang327@163.com (K.W.); zhaoyiguang@126.com (Y.Z.); xuemeinan@126.com (X.N.); chen_ruipeng@yeah.net (R.C.); 2 College of Animal Science and Veterinary Medicine, Tianjin Agricultural University, Tianjin 300384, China

**Keywords:** milk extracellular vesicles, inflammatory bowel disease, gut microbiota, mRNA, lncRNA, circRNA

## Abstract

Harboring various proteins, lipids, and RNAs, the extracellular vesicles (EVs) in milk exert vital tissue-specific immune-protective functions in neonates via these bioactive cargos. This study aims to explore the anti-inflammatory effects of bovine milk-derived EVs on a dextran sulfate sodium (DSS)-induced colitis model and to determine the underlying molecular mechanisms. Sixty C57BL/6 mice were divided into the NC group (normal control), DSS group (DSS + PBS), DSS + LOW group (DSS + 1.5 × 10^8^ p/g EVs), DSS + MID group (DSS + 1.5 × 10^9^ p/g EVs), and DSS + HIG group (DSS + 1.0 × 10^10^ p/g EVs). Histopathological sections, the gut microbiota, and intestinal tissue RNA-Seq were used to comprehensively evaluate the beneficial functions in mitigating colitis. The morphology exhibited that the milk-derived EVs contributed to the integrity of the superficial epithelial structure in the intestine. Additionally, the concentrations of IL-6 and TNF-α in the colon tissues were significantly decreased in the EVs-treated mice. The abundances of the *Dubosiella*, *Bifidobacterium*, *UCG*-*007*, *Lachnoclostridium*, and *Lachnospiraceae* genera were increased in the gut after treatment with the milk-derived EVs. Additionally, the butyrate and acetate production were enriched in feces. In addition, 1659 genes were significantly down-regulated and 1981 genes were significantly up-regulated in the EVs-treated group. Meanwhile, 82 lncRNAs and 6 circRNAs were also differentially expressed. Overall, the milk-derived EVs could attenuate colitis through optimizing gut microbiota abundance and by manipulating intestinal gene expression, implying their application potential for colitis prevention.

## 1. Introduction

Inflammatory bowel disease (IBD), which includes Crohn’s disease and ulcerative colitis, has become a worldwide disease concern with increasing incidence [1]. IBD is believed to be a chronic mucosal inflammatory response that is caused by the combination of genetic susceptibility and environmental factors [2]. Regulating the gut microbiota could improve susceptibility to IBD by producing bioactive metabolites that effect immune activity and epithelial function [3]. Individuals with IBD are associated with intestinal microbiota disorders and a decreased content of short-chain fatty acids (SCFAs) metabolites [4]. Diet is considered to be one of the biggest potential modulators of diversity in the intestinal microbiome and can act as a therapeutic method for treating IBD [5].

Extracellular vesicles (EVs), which have a biomolecular lipid bilayer nanostructure are abundant bioactive ingredients in bovine milk [6]. The prime function of EVs is that they can directly enter the circulating blood and can transport diverse cellular components such as miRNA, circRNA, lncRNA, and tRNA to the target cells, regulating gene expression in cross species tissues [6]. Tong et al. (2020) reported that milk-derived EVs improved the expression of intestinal barrier functions and immune-related genes in mice [7]. The oral gavage of milk EVs was beneficial for preventing experimental necrotizing enterocolitis by increasing the number of goblet cells and endoplasmic reticulum function [8]. Moreover, another role of EVs is that partial EVs can resist the harsh conditions of the gastrointestinal tract and can enter the large intestine and shape the gut microbiota in healthy mice [9,10,11]. Our previous study also demonstrated that the EVs from milk increased the abundance of the “beneficial” microbe *Akkermansia* and decreased the level of the “harmful” microbe *Desulfovibrio* in healthy mice [11]. Therefore, the EVs from milk might be suitable as a functional food to exert an anti-inflammatory role.

Considering their potential role in regulating the expression of immune-related genes and in shaping the gut microbiota, we hypothesized that EVs could alleviate intestinal inflammation. IBD has been widely employed using dextran sulfate sodium (DSS)-induced colitis in mice because of its simplicity, reproducibility, and rapidity [12]. In this study, the anti-inflammatory effect of the oral administration of EVs in alleviating chronic DSS-induced experimental colitis models was examined. Furthermore, we investigated the underlying mechanisms of the therapeutic effects of EVs in regulating the gut microbial community and the transcriptomic landscape to facilitate IBD recovery.

## 2. Materials and Methods

### 2.1. Isolation and Characterization of Milk EVs

The details of how the EVs from the milk of Holstein cows were prepared were described in our previous study [11]. Briefly, the milk-derived EVs were isolated by means of differential centrifugation and ultracentrifugation and were identified by transmission electron microscopy, nanoparticle tracking analysis, and Western blot analysis.

### 2.2. Dextran Sodium Sulfate (DSS)-Induced Colitis

All animal care and experimental procedures strictly followed the guidelines for Care and Use of Laboratory Animals of the Chinese Academy of Agricultural Sciences and were approved by the Animal Ethics Committee of the Chinese Academy of Agricultural Sciences (Beijing, China; approval number: IAS-2020-108).

Specific-pathogen-free (SPF) mice came from the same vendor (SPF Biotechnology Co., Ltd., Beijing, China), were of the same strain (C57BL/6), and were of the same age (six weeks). A total of 60 mice were randomly assigned to 12 cages, with five mice in each cage, and fed over a two-week adaption period before the experiment began. Then, the five cohoused mice in each cage were randomly divided into five groups: normal control (NC), DSS + PBS(DSS), DSS + (1.0 × 10^10^ particles/g (p/g) body weight (BW), DSS + HIG), DSS + 1.5 × 10^9^ p/g BW (DSS + MID), and DSS + 1.5 × 10^8^ p/g BW (DSS + LOW). All of the mice had free access to food and water, and the room had a 12 h light–dark cycle (21 ± 2 °C with a relative humidity of 40–60%).

**Figure 1:**
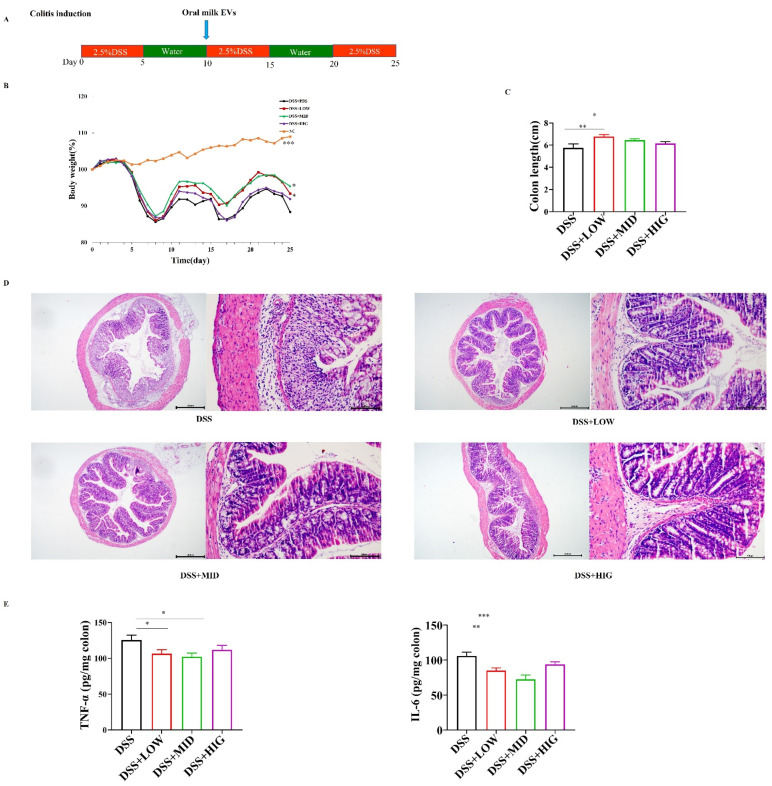
Oral administration of milk-derived EVs ameliorated dextran sulfate sodium (DSS)-induced chronic colitis in mice. (**A**) Experimental design for DSS-induced chronic colitis and oral gavage of EVs. Three dosages of EVs (LOW, MID, and HIG) were orally administered 10 days after the initiation of the experiment. (**B**) The percentage of body weight change. (**C**) Colon length change. (**D**) Representative images of colon tissues stained with hematoxylin and eosin (H&E), scale bar = 500 μm (left) and 100 μm (right). (**E**) Concentrations of two representative pro-inflammatory cytokines in the colon: TNF-α (left) and IL-6 (right). Data were presented as mean ± SEM (*n* = 12 per group); * *p* < 0.05, ** *p* < 0.01, *** *p* < 0.001.

Experimental chronic colitis was induced by repetitively administering 2.5% DSS (molecular weight 36,000–50,000 kDa; MP Biomedicals) for 5 d and then purified water for 5 d alternately in a single 25 d period (Figure 1A), which was determined based on previous studies [13,14]. Animal BW, rectal bleeding, and food and water intake were monitored daily. After treatment, all of the mice were sacrificed under general anesthesia to harvest the blood, colon, gut content, and fecal samples. The colon tissues were cut into three portions. The distal portion was used for histological analysis, the middle portion was used for RNA extraction, and the proximal portion colon was used for the analysis of the pro-inflammatory cytokines.

### 2.3. Histological Evaluation

Neutral formalin (10%) was used to fix the proximal colon samples at 4 °C overnight. The formalin-fixed colon samples were embedded in paraffin and sectioned to be 10 μm in thickness. Subsequently, the sections were stained with hematoxylin and eosin (H&E) and pictured by means of histopathological analysis (Olympus, Tokyo, Japan).

### 2.4. Enzyme-Linked Immunosorbent Assay (ELISA)

A portion of the frozen colon samples was weighed and put into 900 mL PBS followed by ultrasonic trituration and centrifugation at 12,000× *g* at 4 °C for 15 min to obtain colon homogenate. The colon concentrations of IL-6 (Dakewe, Cat. No. 1210602) and TNF-α (Dakewe, Cat. No. 1217202) were quantified by ELISA Kits according to the manufacturer’s recommendations.

### 2.5. Sequencing of Gut Microbiota

Total microbial DNA extraction of the colonic contents or fecal samples was performed using QIAamp Fast DNA Stool Mini Kits (Qiagen, catalog number:51604, Hilden, Germany). The integrity and concentration of the isolated DNA were identified by agarose gel electrophoresis (1%) and NanoDrop2000, respectively.

The DNA samples acted as the template for the PCR amplification of the bacterial 16S rRNA genes using the primers 338F (5′-ACTCCTACGGGAGGCAGCAG-3′) and 806R (5′-GGACTACHVGGGTWTCTAAT-3′) designed for the V3-V4 region. Then, the library of the PCR amplification products was built using the Illumina Miseq platform at Majorbio Bio-Pharm Technology Co., Ltd. (Shanghai, China).

According to the unique barcode, the 16S rRNA gene sequencing data were classified into different samples. Then, the data were merged, filtered, and further allocated to operational taxonomic units (OTUs) with 97% similarity. Representative data were assigned taxonomy based on the RDP classifier Bayesian algorithm in the Silva database. The MOTHUR program was used to calculate the alpha diversity indices and the rarefaction curves of the gut microbiota according to the OTU levels. The differences in the bacterial community were determined using the LEfSe (linear discriminant analysis (LDA) effect size) method. PICRUSt (phylogenetic investigation of communities by reconstruction of unobserved states) was conducted to predict the functions of 16S rRNA amplicon sequencing.

### 2.6. Short-Chain Fatty Acids (SCFAs) Quantification

The concentrations of acetate, propionate, and butyrate in the fecal samples were measured by gas chromatography. Briefly, the fecal pellets and colonic contents were dissolved in distilled water and homogenized by continuous vortexing. Then, the samples were centrifuged at 10,000× *g* and 4 °C for 10 min to obtain clear supernatant, which was then mixed with a 25% metaphosphoric acid solution in a 10:1 (*v*/*v*) ratio. The tubes were kept at room temperature for 30 min and then centrifuged at 10,000× *g* and 4 °C for 15 min. Finally, the supernatant was harvested and analyzed by Agilent 5975C gas chromatography (Agilent Technologies, Inc., Palo Alto, CA, USA).

### 2.7. RNA Sequencing and Analysis

The total RNA in the colonic tissues was extracted using TRIzol (Invitrogen, Waltham, MA, USA). RNA quality and quantity were determined by NanoDrop2000, 1% agarose gel electrophoresis, and Agilent 2100 (Agilent, Santa Clara, CA, USA). The isolated RNAs were treated using an rRNA Removal Kit (Illumina, San Diego, CA, USA) following the manufacturer’s protocols to remove the ribosomal RNA. The remaining RNA was fragmented into 250–300 bp fragments using a fragmentation buffer and were reverse transcribed into first strand cDNA with random hexamers. The second strand cDNA was synthesized using DNA polymerase I, dNTPs (dATP, dUTP, dCTP and dGTP), and buffer, and AMPure XP beads were then applied to purify the cDNA fragments. Next, the purified double-strand cDNA fragments were end repaired, had poly(A) added, and were ligated to Illumina sequencing adaptors. After the cDNA fragments were purified using the AMPure XP system, the 250–300 bp fragments were preferentially selected and degraded by means of Uracil-N-Glycosylase followed by PCR amplification and Illumina sequencing using PE150 platform (paired-end 150 nt) sequencing to obtain 12 G raw data.

After sequencing, the raw data was subjected to a series of pretreatments, including quality control, mapping and assembly, and the identification and annotation of novel transcripts. There were four steps that were implemented to identify the lncRNA from the assembled transcripts. First, the low-expressed transcripts with less than 0.5 FPKM (Fragments Per Kilobase of transcript per Million mapped reads) were removed. Second, the transcripts that with less than 2 exons and shorter than 200 bp were also removed. Third, the Coding Potential Calculator (CPC), Coding-Non-Coding Index (CNCI), and Pfamscan (PFAM) databases were used to remove the transcripts with protein-coding ability. Finally, Cuffcompare was used to remove the transcripts that were located within the 1 kb flanking regions of an annotated gene. The transcript abundance was quantified as FPKM using the Cuffdiff program. The R package edgeR was used to analyze the differentially expressed mRNA and lncRNA, with *p* < 0.05 and log_2_-transformed fold-change (|log_2_FC|) of >2.

For the circRNAs, the raw data were filtrated to obtain high-quality clean data, which were further aligned with the reference genome. The circRNAs were detected and identified using find_circ (https://github.com/marvin-jens/find_circ) (accessed on 3 November 2020) and CIRI2 (https://sourceforge.net/projects/ciri/) (accessed on 3 November 2020). Briefly, the potential circRNAs in all of the unaligned back-spliced junction reads were screened, and the identified circRNAs that were expressed in at least two samples were qualified for further analysis. Then, the raw counts were normalized using TPM to determine the read counts, and a differential expression analysis was conducted using the DESeq R package. If *p* < 0.05 and |log_2_FC| > 2, then the circRNAs were considered to be differentially expressed. The target sites of the MiRNAs in of the exons in the circRNAs were predicted using miRanda (miRanda 3.3.0), and the circRNAs–miRNAs–gene network was constructed using cytoscape software (Cytoscape 3.8.0).

### 2.8. Functional Enrichment Analysis

LncRNA can trans-regulate distant target genes because they have the same expression pattern. Thus, the Pearson correlations coefficients (PPCs) between the coding genes and lncRNAs were calculated, and the co-expressed lncRNA–mRNA was selected to have |PCC| > 0.95 and *p* < 0.05. The functions of the differentially expressed mRNA and lncRNA co-expression were analyzed using the Gene Ontology (GO) and Kyoto Encyclopedia of Genes and Genomes (KEGG) pathways.

### 2.9. RT-PCR

RT-PCR was conducted to verify the transcriptome results. Reverse transcription was conducted using the PrimeScript RT Master Mix kit (cat. no. RR036a, TaKaRa, Kusatsu, Japan) following the manufacturer’s instructions. The primers that were used in this study were designed and had their specificity checked using the Primer Premier 6 and Primer BLAST tools in NCBI, respectively, and then synthesized by Sangon biotech (Beijing, China) (Table S1). GAPDH acted as the house-keeping gene for normalization. The RT-PCR reactions were treated using TB Green^®^ Premix Ex Taq™ II (cat. no. RR820a, TaKaRa, Japan) on the QuantstudioTM 7 flex system (ABI Q7 Flex 384well, life technologies, Carlsbad, CA, USA). The reaction system contained 5 μL TB Green Premix Ex Taq II (Tli RNaseH Plus) (2×), 0.8 μL of 10 μΜ forward and reverse primers, 0.2 μL ROX Reference Dye (50×), 1 μL template cDNA, and 3 μL nuclease free water. The PCR cycling conditions were as follows: initial denaturation at 95 °C for 30 sec followed by 40 cycles at 95 °C for 5 sec and 60 °C for 34 sec, a dissociation stage at 95 °C for 15 sec, 60 °C for 1 min, and 95 °C for 15 sec. The relative expression was calculated using the formula 2−ΔΔCt, where ∆∆Ct = (Ct_Target_ − Ct_GAPDH_) EVs − (CT_Target_ − Ct _GAPDH_) PBS.

### 2.10. Statistical Analysis

The data were presented as the mean ± SEM and depicted using GraphPad Prism 7.0 (GraphPad Software, Inc., San Diego, CA, USA). The significance levels of the colonic length and SCFA content were determined using one-way analysis of variance (ANOVA) (SPSS 21, IBM, Chicago, Armonk, NY, USA). The RT-PCR results were analyzed using unpaired Student’s t-tests (SPSS 21, IBM, Armonk, NY, USA). A significant difference was declared at *p* < 0.05.

## 3. Results

### 3.1. Milk EVs Ameliorated Colitis Symptoms

To study the therapeutic efficacy of EVs against IBD, we established chronic colitis murine models using DSS. Compared to the DSS group, the administration of oral EVs in the LOW and MID groups significantly decreased BW loss and protected the mice against colon shortening (Figure 1B,C). However, there were no differences in BW loss and colon length between the DSS group and the HIG group (Figure 1B,C). An investigation of the intestinal morphology exhibited that the milk-derived EVs-treated groups had a more integrated superficial epithelial structure compared to the DSS group (Figure 1D). In addition, the TNF-α and IL-6 concentrations in the colon tissues were significantly decreased in the DSS-treated mice in response to increasing oral EVs supplementation (Figure 1E). Collectively, the milk-derived EVs effectively alleviated clinical symptoms and colonic damage in DSS-induced colitis in mice.

### 3.2. Milk EVs Benefited Gut Microbiota and SCFAs Production

To elucidate whether the oral gavage of milk-derived EVs could alleviate DSS-induced colitis by altering gut microbial community composition, we further investigated the fecal microbiota. In our study, a total of 3,354,738 raw reads and 1,677,369 high-quality sequences were obtained from 24 fecal samples, with an average read length of 415 bases. After screening with 97% sequence similarity, 531 OTUs were generated across all of the samples (Figure S1A). The Shannon, Simpson, ACE, and Chao α-diversity were not influenced by the treatments, indicating that the milk-derived EVs did not significantly alter the diversity and richness of the gut microbiota (Table S2). To assess overall differences in the β-diversity, the unweighted UniFrac distance metric matrices were generated from all of the samples based on the OUT level (Figure S1B).

**Figure 2:**
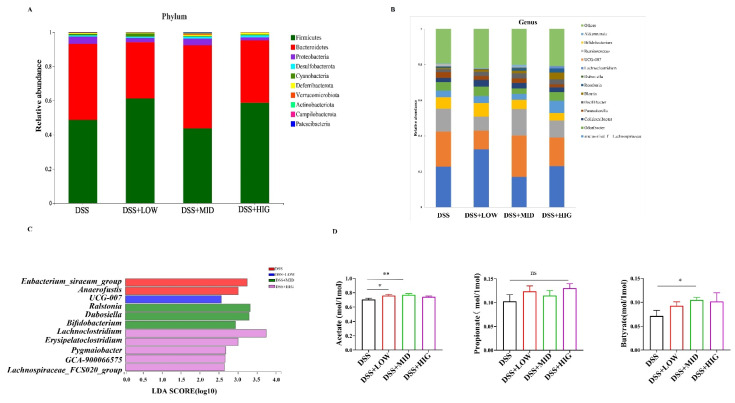
Gut microbiota and short-chain fatty acids in response to the oral administration of milk-derived EVs in the DSS-induced colitis model. (**A**) The composition of the gut microbiota at the phylum level. (**B**) The composition of the predominant gut microbiota at the genus level. (**C**) Linear discriminant analysis (LDA) score derived from LEfSe analysis of the bacterial community at the genus level. An LDA score >2.5 was considered significant. (**D**)The concentrations of fecal acetate, propionate, and butyrate. Data are presented as mean ± SEM (*n* = 6 per group); * *p* < 0.05, ** *p* < 0.01, ns *p* ≥ 0.05.

At the phylum level, ten bacterial phyla were identified across all of the fecal samples. Firmicutes and Bacteroidetes were the two predominant phyla, and when combined, they accounted for over 85% (Figure 2A). At the genus level, 126 bacterial genera were detected in all of the samples. Lachnospiraceae_NK4A136_group (23.86%), Bacteroides (17.32%), norank_f__Muribaculaceae (11.26%), unclassified_f__Lachnospiraceae (4.49%), and Odoribacter (4.48%) were the dominant genera (Figure 2B). LEfSe was used to analyze the different bacteria between the DSS group and the three EVs groups. An LDA score of >2.5 was considered as a significant difference. The genera Dubosiella and Bifidobacterium were significantly enriched in the MID EVs group. The genus UCG-007 was significantly enriched in the LOW EVs group. Additionally, the genera Lachnoclostridium and Lachnospiraceae_FCS020_group were significantly enriched in the HIG EVs group (Figure 2C).

To further speculate on the functions of the gut microbiota, 16S rRNA gene sequences were used to indirectly infer the microbiome gene composition by PICRUSt analysis (Figure S2). At the superclass level, 18,200,939 genes were involved in metabolism, 7,197,537 genes were related to genetic information processing, 6,052,314 genes participated in environmental information processing, 1,603,318 genes were included in cellular processes, 273,337 genes participated in human diseases, and 5,421,552 genes were unclassified. At the class level, a total of 37 KEGG pathways were noted. The most abundant functional categories were the membrane transport, carbohydrate metabolism, amino acid metabolism, and replication and repair categories. These findings suggested that the milk-derived EVs could regulate metabolic pathways, genetic information processing, and environmental information processing in DSS-induced chronic colitis mice and could restore maladjusted functions.

SCFAs are the primary products of the dietary fiber fermentation that is carried out by the gut microbiota and play an important role in intestinal health and anti-inflammatory status. The results of three main SCFAs (acetate, propionate, and butyrate) in this study were shown in Figure 2D. Compared to the DSS group, the acetate concentrations were significantly increased in the LOW and MID EVs groups (*p* < 0.05), while the propionate concentrations were not affected by the treatments using milk-derived EVs (*p* > 0.05). Moreover, the butyrate concentrations were significantly increased in the MID EVs group (*p* < 0.05) and numerically increased in the LOW and HIG groups (*p* > 0.05) compared to the DSS group.

### 3.3. EVs Changed mRNA and Non-Coding RNA Expression

Based on the results of the colitis symptoms and the gut microbiota, we found that the middle EVs treatment group showed the most significant effects. Therefore, the DSS group (DSS + PBS) and DSS + MID group (DSS + MID-dosage EVs) were chosen for transcriptome sequencing. A total of 1,123,431,382 raw reads (168.53 Gb) were generated in all of the libraries. The Q30 of all of the reads that were distributed from 93.52% to 94.72% and the average GC content was 53.20% (Table S3). These results indicated that the quality of the sequencing data was highly reliable. A total of 8552 lncRNAs were identified using three programs: CNCI, CPC, and PFAM (Figure S3A). A total of 3878 lncRNAs, including 1806 sense lncRNAs, 1469 long intergenic noncoding RNAs (lncRNAs), and 603 anti-sense lncRNAs, were identified (Figure S3B). A Venn diagram presents the results of the mRNAs and lncRNAs that were expressed in the DSS and MID groups (Figure S3C,D). In total, 2422 mRNAs and 2334 lncRNAs were expressed specifically in the MID group.

#### 3.3.1. EVs Altered Gut Gene Expression

**Figure 3:**
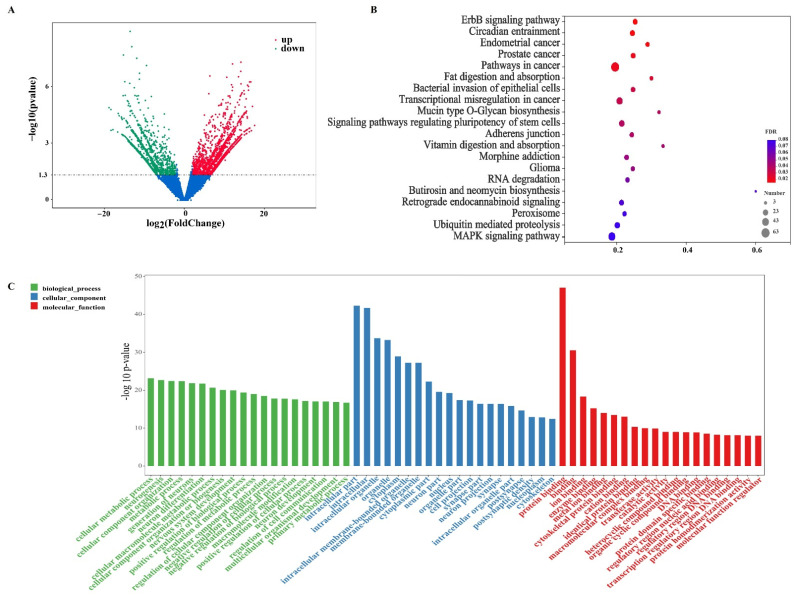
Volcano plots, KEGG pathways, and GO analysis of differentially expressed mRNAs in response to the oral gavage of milk-derived EVs in the DSS-induced murine colitis model. (**A**): Volcano plots of differentially expressed mRNAs were formed with the log_2_ ratio and −log_10_ adjusted *p*-values for the DSS group versus the DSS + MID group, where log_2_ (FC) > 2 and *p* < 0.05 represent up-regulation, log_2_(FC) < −2 and *p* < 0.05 represent down-regulation, and the probes in blue represent significance (*p* < 0.05) but with small fold changes (log_2_(FC) > −2 and <2). (**B**): Kyoto Encyclopedia of Genes and Genomes (KEGG) functional analysis revealed the biological pathways that were enriched in the differentially expressed mRNAs. The Y-axis label is the pathway, and the X-axis label is the rich factor (rich factor = the number of differentially expressed genes enrichment in the pathway/ all genes). (**C**): The enriched GO terms. *n* = 6 samples/group.

Based on cutoff values of log_2_ FC > 2(up-regulated), log_2_ FC < −2(down-regulated), and *p* < 0.05, we identified numerous differentially expressed (DE) mRNAs (Figure 3A, Table S4). Compared to the DSS group, the EVs group had 3640 DE genes (1659 up-regulated and 1981 down-regulated) in the DSS-induced colitis mice. Furthermore, RT-PCR was used to verify the validity of the transcriptome sequencing, and the results were confirmed with the transcriptome data (Figure S4).

As shown in Figure 3C, the top 20 enriched GO terms for the biological process (BP), cellular component (CC), and molecular function (MF) were identified. The top 5 significantly enriched GO terms were protein binding, intracellular parts, intracellular, intracellular organelle, and organelle. Meanwhile, 13 KEGG pathways were significantly annoted (Figure 3B), including the ErbB signaling pathway, circadian entrainment, endometrial cancer, prostate cancer, pathways in cancer, fat digestion and absorption, bacterial invasion of epithelial cells, transcriptional misregulation in cancer, mucin type O-Glycan biosynthesis, signaling pathways regulating the pluripotency of stem cells, adherens junction, vitamin digestion and absorption, and morphine addiction. Among them, four KEGG pathways, namely the ErbB signaling pathway, bacterial invasion of epithelial cells, mucin type O-Glycan biosynthesis, and adherens junction were associated with intestinal inflammation; two KEGG pathways, namely vitamin digestion and absorption and fat digestion and absorption, were related to nutrition metabolism.

#### 3.3.2. EVs Changed Gut lncRNAs Expression

**Figure 4:**
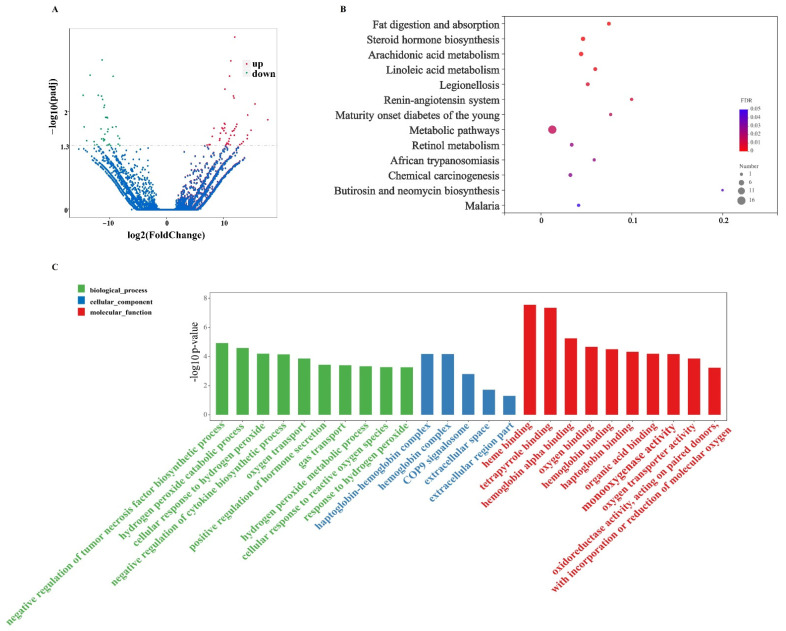
Volcano plots, KEGG pathways, and GO analysis of differentially expressed lncRNAs in response to the oral gavage of milk-derived EVs in the DSS-induced murine colitis model. (**A**): Volcano plots of differentially expressed lncRNAs were formed with the log2 ratio and −log10 adjusted *p*-values for the DSS group versus the DSS + MID group, where log2 (FC) > 2 and *p* < 0.05 represent up-regulation, log2(FC) < −2 and *p* < 0.05 represent down-regulation, and the probes in blue represent significance (*p* < 0.05) with small fold changes (log2(FC) > −2 and <2). (**B**): Kyoto Encyclopedia of Genes and Genomes (KEGG) analysis revealed the biological pathways that were enriched in the differentially expressed lncRNAs. The Y-axis label is the pathway, and the X-axis label is the rich factor (rich factor = the number of differentially expressed lncRNAs enrichment in the pathway/ all lncRNAs). (**C**): The enriched GO terms. *n* = 6 samples/group.

The EVs significantly up-regulated 46 lncRNAs and down-regulated 36 lncRNAs in the murine model of colitis, and |log_2_ FC| > 2 and *p* < 0.05 was used as the cutoff level (Figure 4A, Table S5). The functional annotations of the lncRNAs were mostly based on co-expression (trans-regulation). According to the |PCC| > 0.95 and *p* < 0.05 standard, 150 DE mRNAs were found to be co-expressed with the DE lncRNAs. The top three significantly changed GO terms related to the milk-derived EVs were heme binding, tetrapyrrole binding, and the negative regulation of the tumor necrosis factor biosynthetic process (Figure 4C). We analyzed the KEGG pathways of the trans lncRNAs target genes and found that 12 KEGG pathways were significantly enriched (Figure 4B). Additionally, among them, six KEGG pathways, namely fat digestion and absorption, steroid hormone biosynthesis, arachidonic acid metabolism, linoleic acid metabolism, metabolic pathways, and retinol metabolism, were related to nutrition metabolism.

#### 3.3.3. EVs Changed Gut circRNAs Expression

**Figure 5:**
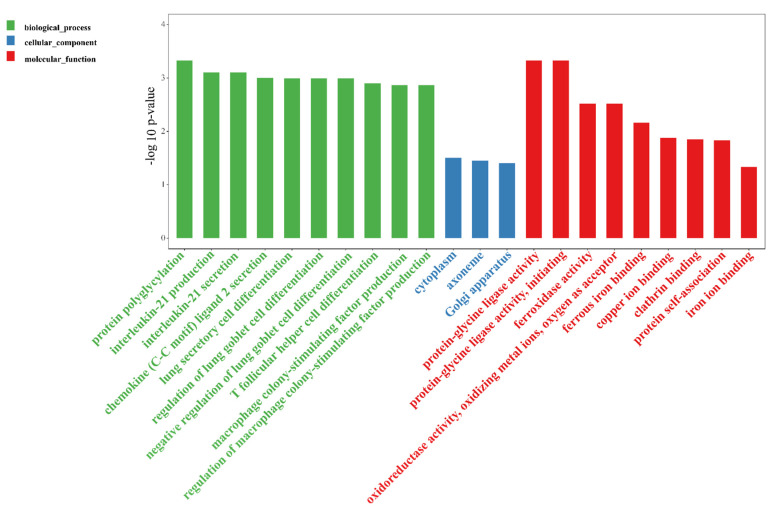
GO enrichment of the differentially expressed circRNAs in response to the oral gavage of milk-derived EVs in the DSS-induced murine colitis model. *n* = 6 samples/group.

**Figure 6:**
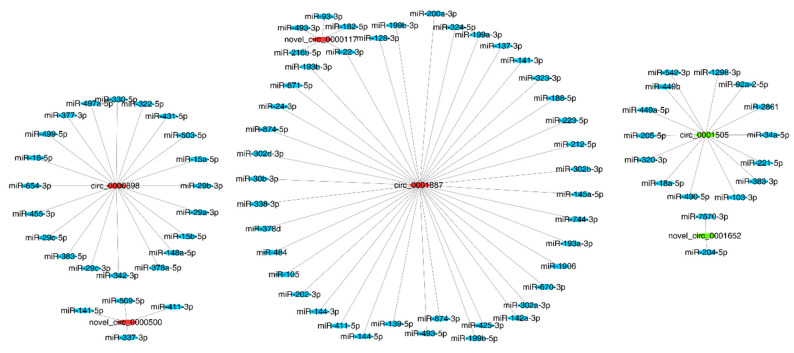
Overview of the differentially expressed circRNAs and miRNAs binding sites, and the figures were drawn using Cytoscape software. *n* = 6 samples/group.

To profile the circRNA landscape in the colon tissues between the DSS and DSS + MID groups, we identified 1225 circRNAs in 12 libraries. The number of co-expressed circRNAs in the two groups were 512, while 450 and 236 were specifically expressed in the DSS and MIDg groups, respectively (Figure S3E). However, only six DE circRNAs were found in the two groups, of which two circRNAs were up-regulated, and four circRNAs were down-regulated in the MID group. The up-regulated circRNAs were circRNA_0001505 and circRNA_0001652, and the down-regulated circRNAs were circRNA_0000898, circRNA_0001887, circRNA_0000117, and circRNA_0000500, respectively (Table 1). In addition, circ_0000117 and circ_0000500 possessed internal ribosome entry site (IRES) elements, and their IRES scores were 0.780 and 0.770, respectively. Furthermore, the GO analysis showed that the DE circRNAs were preferentially enriched in terms of biological process and immune-related functions (Figure 5). To better understand the function of circRNAs, we utilized Cytoscape software to predict the miRNAs using the six DE circRNAs. The results were presented in Figure 6. A total of 83 circRNAs–miRNAs pairs were obtained, from which circ_0001887 could predict more miRNAs. These circRNAs and miRNAs might be the potential targets of the EVs in alliviating colitis.

## 4. Discussion

Gut microbial dysbiosis is closely associated with the occurrence and development of colitis. In the current study, we found that the oral gavage of milk-derived EVs protected mice against the gut microbiota dysbiosis during DSS-induced colitis. *Bifidobacterium*, a common probiotic, is directly involved in the immunological regulation process of inflammation, and produced cytokines against pathogens invasion [15]. In addition, *Bifidobacterium* also has the ability to prevent or attenuate intestinal inflammation [15,16]. Previous studies have demonstrated that the bacteria *Dubosiella* and *Lachnoclostridium* decreased dramatically in animal models of colitis, suggesting that they might be beneficial in attenuating colitis [17,18,19]. The abundance of *Dubosiella* was positively correlated with butyrate production [18]. The genus *Lachnoclostridium* could utilize monosaccharides and disaccharides to produce acetate [20]. Acetate can effectively stabilize intestinal homeostasis via anti-inflammatory and immunosuppressive functions [21,22]. Butyrate promotes the development of intestinal epithelial cells, inhibits the release of proinflammatory cytokines, and improves mucosal barrier function [23]. Taken together, our study suggests that EVs supplementation could improve the abundance of *Bifidobacterium*, *Dubosiella*, and *Lachnoclostridium* and increase the concentrations of acetate and butyrate, which might explain the effect of EVs in alleviating colitis.

A study has demonstrated the importance of host gene regulation in IBD prevention [2]. Our study explored the mRNAs profile in response to oral EVs in DSS-induced colitis. The supplementation of EVs increased the expression of Ptprs (protein–tyrosine phosphatase sigma), Slc6a4(serotonin reuptake transporter mRNA), Defb1 (defensin beta 1), Tafa4 (Tafa chemokine like family member 4), Serpina3k(serine peptidase inhibitor, clade A, member 3K), Cfd(complement factor D), Usp44 (ubiquitin specific peptidase 44), and CD69 genes and decreased the expression of the Trim27 (tripartite motif containing 27), Lamc2(Laminin subunit γ2), and Traf3 (TNF receptor associated factor 3) genes in DSS-induced colitis. Ptprs, a receptor-type protein tyrosine phosphatase is a susceptibility gene for ulcerative colitis, and the Ptprs-knockout mice spontaneously suffered from mild colitis [24]. The Slc6a4 gene could encode the serotonin reuptake transporter, which is the only high-affinity and competent transporter of serotonin [25]. Slc6a4 expression was significantly down-regulated in intestinal inflammation [25]. Defb1 could increase the abundance of bacteria with anti-inflammatory effects and consequently attenuate inflammatory disease [26,27]. Tafa4, as a new therapeutic target gene for inflammation, could improve the anti-inflammatory effects of macrophages, and prevent fibrosis after tissue injury [28]. Serpina3k, an important anti-inflammatory gene, has been reported to be down-regulated during inflammation [29]. Cfd is characterized by maintaining gut homeostasis [30]. Usp44 is found to regulate the Tregs function by preventing FOXP3 degradation [31]. Meanwhile, CD69 has the ability to enhance the immunosuppressive function of Tregs and to alleviate colitis by improving IL-10 production [32]. Trim27 deficiency could decrease the levels of pro-inflammatory cytokines (IL-6, TNF-α, and IL-17A) in the colonic mucosa and suppress the DSS-induced colitis [33,34]. Lamc2 and Traf3 are usually overexpressed in the colitis, and both of them would further aggravate inflammation [35,36]. In addition, chronic inflammation potentially develops into colitis-associated colorectal cancer (CAC). In the current study, we also observed the differential expressions of several genes closely related to CAC. The Ptprt gene (protein tyrosine phosphatase receptor-T) is a tumor suppressor, and Ptprt-knockout mice are more susceptible to CAC [37]. The Pkn2 (protein kinase N2) gene has the ability to inhibit tumor-associated macrophages polarization and tumor growth [38]. However, Dixdc1 acts as a positive regulator in the Wnt pathway, and its overexpression could promote the proliferation and invasion of colon cancer cells [39]. Dach1(Dachshund homologue 1) is a tumor promoter that is highly expressed in all stages of CAC [40]. Here, we found that the EVs significantly increased Ptprt and Pkn2 expression but decreased Dixdc1 and Dach1 expression in the murine models of chronic inflammatory disease. Above all, these results suggested that the protective effects of EVs on colitis could be related to the up-regulation of anti-inflammatory genes and the down-regulation of pro-inflammatory genes.

Apart from the mRNAs, the dysregulation of lncRNAs expression is also involved in IBD [41]. However, the functions of most lncRNAs are not fully understood. Recent evidence documented that they could regulate the expression of protein-coding genes [42]. In our study, differentially expressed lncRNAs were used to predict their target genes, and we found that EVs significantly up-regulated the expression of the Slc9a8 (solute carrier family 9 member A8), Tnfaip8 (TNF-α-induced protein 8), and Crept (Cell cycle-related and expression elevated protein in the tumor) and down-regulated the expression of the Sema5a (Semaphorin 5A) and Morrbid (myeloid RNA regulator of BCL2L11 induced cell death) genes in DSS-treated mice. Slc9a8, also known as Nhe8, maintains intestinal mucosal integrity by modulating the functions of the goblet and Paneth cells; moreover, Slc9a8 deficiency is hypersensitive to DSS challenge [43]. Tnfaip8 is critical for the maintenance of intestinal homeostasis, and Crept is required for the regeneration of intestinal epithelial cells. Therefore, both of them are beneficial to the attenuation of colitis [44,45]. Sema5a, an axon guidance cue, could induce the expression of pro-inflammatory genes (TNF-α and IL-8) [46]. In addition, lncRNA Morrbid, which was only identified recently, plays an important role in the onset and progression of the disease [47]. In the current study, the KEGG pathway analysis found that the differentially expressed lncRNAs were mainly associated with fat digestion and absorption, steroid hormone biosynthesis, arachidonic acid metabolism, linoleic acid metabolism, metabolic pathways, and retinol metabolism. The enriched pathways indicated that EVs-induced regulation of lipid metabolism may have a close relationship with the effects of inhabiting intestinal inflammation. However, the associated mechanism still requires further investigation.

## 5. Conclusions

The oral administration of milk-derived EVs attenuated intestinal inflammation in DSS-induced chronic colitis models. The EVs reversed gut microbiota dysbiosis to protect the mice against DSS-induced colitis, particularly through the increments in the relative abundance of the SCFA-producing bacteria. Furthermore, the EVs effectively up-regulated the expression of anti-inflammatory genes and down-regulated the expression of pro-inflammatory genes. This study provides novel information regarding the biological functions of milk-derived EVs, which might contribute to the development of an effective dietary additive for the treatment of intestinal dysfunction.

## Figures and Tables

**Table 1:** Differentially expressed circRNAs from the DSS and DSS + MID groups.

Circ_id	DSS + MID	DSS	Log2(FC)	*p*-Value
mmu_circ_0000898	0.44	3.15	−3.15	0.03
mmu_circ_0001505	5.28	0.33	3.31	0.01
mmu_circ_0001887	0	2.54	−3.75	0.04
novel_circ_0000117	0.65	5.85	−3.42	0.02
novel_circ_0000500	0	3.92	−4.34	0.01
novel_circ_0001652	2.44	0	3.78	0.04

## Data Availability

All the raw sequences of the transcriptome and microbiome were submitted to the NCBI Sequence Read Archive (SRA1) under accession numbers SRP343830 and SRP348793, respectively.

## Supplementary Materials

The following supporting information can be downloaded at https://www.mdpi.com/article/10.3390/nu14091808/s1, Table S1: List of primer sequences used in the RT-PCR, Table S2: Alpha diversity index of bacterial 16S rRNA gene sequences, Table S3: The results of raw reads after quality control, Table S4: Differentially expressed mRNAs between DSS and DSS + MID groups in the DSS-induced colitis model, Table S5: Differentially expressed lncRNAs between DSS and DSS + MID groups in the DSS-induced colitis model, Figure S1: (A) Effects of milk EVs on the OTUs of microbiota in the colitis model. (B) Hierarchical clustering tree on OUT level, Figure S2: PICRUSt analysis to predict functional profiling of gut microbiota in the colitis model, Figure S3: Summary of lncRNA characteristics, mRNAs expression and circRNAs expression: (A) Screening of the protein’s potential in lncRNAs. (B) The types of lncRNAs. (C) Venn diagram of expressed mRNAs in DSS and DSS + MID groups. (D) Venn diagram of expressed lncRNAs in DSS and DSS + MID groups. (E) Venn diagram of expressed circRNAs in DSS and DSS + MID groups. Figure S4: The verification of differentially expressed mRNAs using RT-PCR.
