# Supplementary material for: Supplementation with Milk-Derived Extracellular Vesicles Shapes the Gut Microbiota and Regulates the Transcriptomic Landscape in Experimental Colitis
Source: Nutrients. 2022 Apr 26;14(9):1808. doi: 10.3390/nu14091808 (PMC9104790; doi:10.3390/nu14091808)
Supplement: Supplementary file 1 [file nutrients-14-01808-s001.zip › Figure S1-S4.pdf]

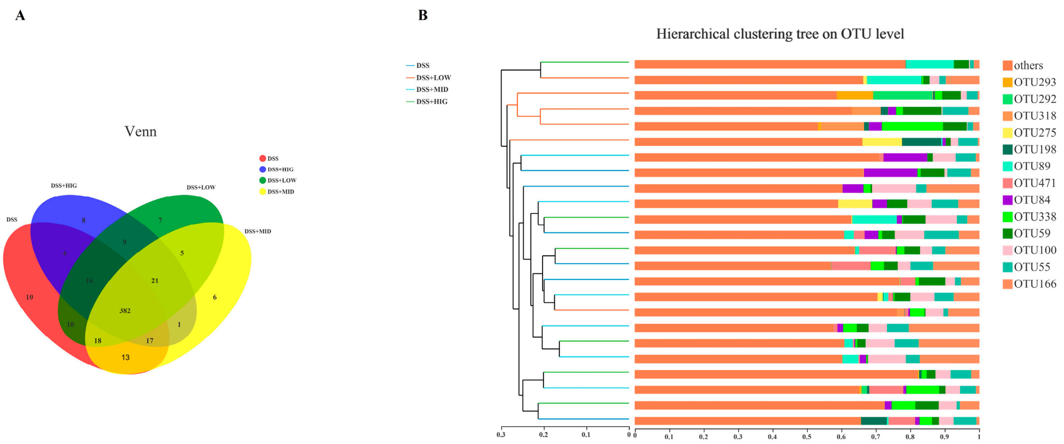

Figure S1. (**A**) Effects of milk EVs on the OTUs of microbiota in the colitis model. (**B**) Hierarchical clustering tree on OUT level

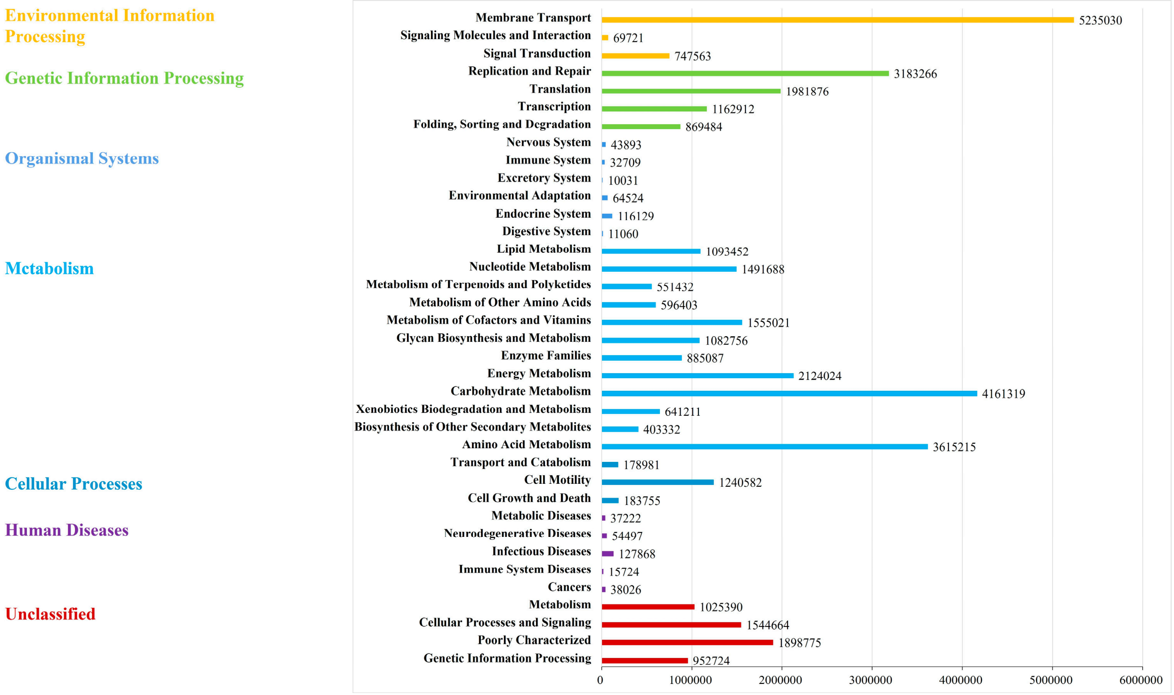

Figure S2. PICRUSt analysis to predict functional profiling of gut microbiota in the colitis model.

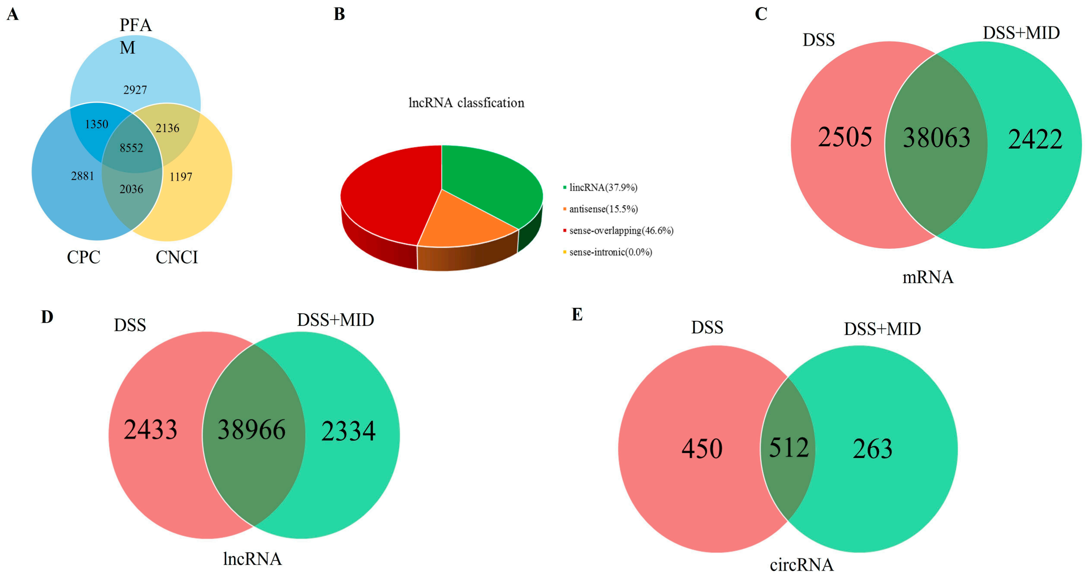

Figure S3. Summary of lncRNA characteristics, mRNAs expression and circRNAs expression: (**A**) Screening of the protein's potential in lncRNAs. (**B**) The types of lncRNAs. (**C**) Venn diagram of expressed mRNAs in DSS and DSS + MID groups. (**D**) Venn diagram of expressed lncRNAs in DSS and DSS + MID groups. (**E**) Venn diagram of expressed circRNAs in DSS and DSS + MID groups.

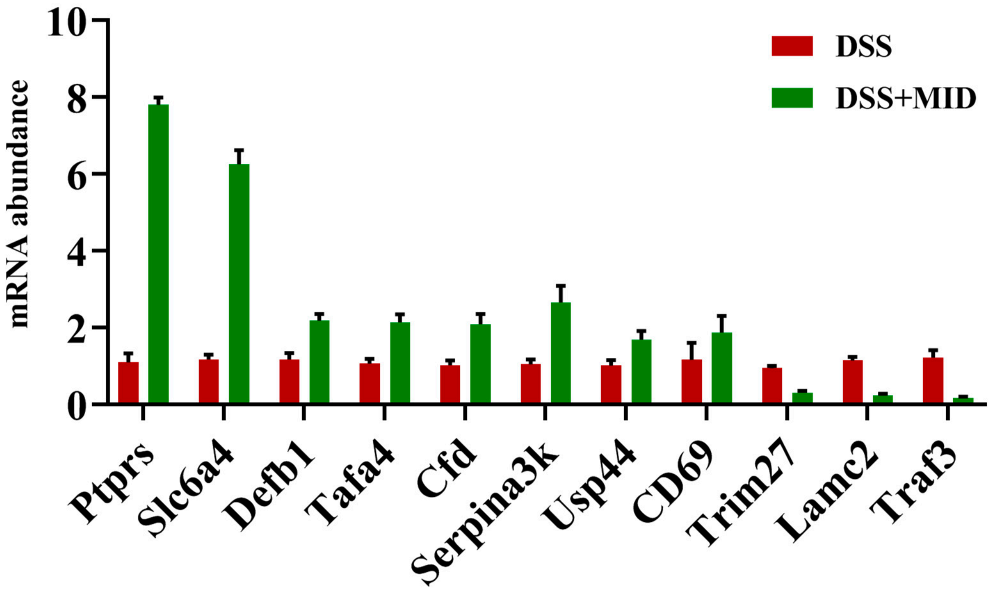

Figure S4. The verification of differentially expressed mRNAs using RT-PCR.
