# Supplementary material for: Supplementation with Milk-Derived Extracellular Vesicles Shapes the Gut Microbiota and Regulates the Transcriptomic Landscape in Experimental Colitis
Source: Nutrients. 2022 Apr 26;14(9):1808. doi: 10.3390/nu14091808 (PMC9104790; doi:10.3390/nu14091808)
Supplement: Supplementary file 1 [file nutrients-14-01808-s001.zip › Table. S1-3.pdf]

Table S1. List of primer sequences used in the RT-PCR.

Genes	Primers	Primer Sequences (5′-3′)
Ptprs	F	CCAAGAACCGCTATGCCAAC
	R	ACACCATCCGCCAGAAGTC
Slc6a4	F	ACCAGCAGACAAGGCAGAG
	R	GCCAATGACAGACAGGAGGA
Defb1	F	GGCATTCTCACAAGTCTTGGA
	R	AGTTGGGCTTATCTGGTTTACA
Tafa4	F	GCAATAAGAACCGCATAGAGGA
	R	GGCACCACCACTTCTCAATC
Serpina3k	F	CAGACTTCCAGCAGCCTACT
	R	AATTCACCAGCACCATCAATGT
Cfd	F	GGACGGATGACGACTCTGT
	R	GGACCCAACGAGGCATTCT
Usp44	F	GGACAGCAGATGAACGAAGG
	R	CAGACCACATGACTTGGAACA
CD69	F	AGGCTTGTACGAGAAGTTGGA
	R	TGGTTGTGGTGGAGAAGAAGT
Trim27	F	AGCATGAGTACCGCCTGTT
	R	TGTGTCTCCAATGTCCTGTAAG
Lamc2	F	CGCCTGGATGACCTCAAGA
	R	CGTAGTGCTCAGAAGAATGGAT

Traf3	F	ACACAGGCTTGCTGGAGTC
	R	CGCTTGTAGTCACGGATCTTC
GAPDH	F	TCAAGCTCATTTCCTGGTATGAC
	R	GGATAGGGCCTCTCTTGCTC

Table S2. Alpha diversity index of bacterial 16S rRNA gene sequences.

**Items**	**DSS**	**DSS+LOW**	**DSS+MID**	**DSS+HIG**	**SEM**	***p*-value**
Shannon	3.794	3.824	3.911	3.720	0.0632	0.781
Simpson	0.0511	0.0472	0.0431	0.0507	0.00361	0.865
ACE	343	320	320	332	6.840	0.591
Chao	351	325	319	339	7.488	0.421

Table S3. The results of raw reads after quality control.

**Sample**	**Raw eads**	**Clean reads**	**Clean bases(G)**	**Error rate(%)**	**Q20(%)**	**Q30(%)**	**GC content (%)**
DSS+MID1	81198336	78805500	11.82	0.03	97.92	94.11	54.65
DSS+MID2	90903036	88020946	13.2	0.03	97.94	94.12	53.71

DSS+MID3	1.05E+08	1.01E+08	15.17	0.03	97.63	93.52	52.8
DSS+MID4	81988624	79747986	11.96	0.02	98.15	94.72	52.61
DSS+MID5	84120420	80504202	12.08	0.03	97.65	93.56	49.83
DSS+MID6	1.07E+08	1.05E+08	15.73	0.03	97.72	93.67	51.05
DSS1	1.03E+08	99744514	14.96	0.03	97.76	93.72	50.42
DSS2	1.06E+08	1.02E+08	15.27	0.03	97.83	93.91	50.46
DSS3	1.05E+08	1.01E+08	15.14	0.03	97.62	93.51	52.94
DSS4	81737170	79338556	11.9	0.03	97.92	94.24	61.29
DSS5	93575622	90054320	13.51	0.03	97.78	93.74	49.47
DSS6	84263602	80662476	12.1	0.03	97.93	94.3	59.12
